# Efficacy and safety of ruxolitinib in patients with newly-diagnosed polycythemia vera: futility analysis of the RuxoBEAT clinical trial of the GSG-MPN study group

**DOI:** 10.1007/s00277-022-05080-7

**Published:** 2022-12-23

**Authors:** Steffen Koschmieder, Susanne Isfort, Dominik Wolf, Florian H. Heidel, Andreas Hochhaus, Philippe Schafhausen, Martin Griesshammer, Denise Wolleschak, Uwe Platzbecker, Konstanze Döhner, Philipp J. Jost, Stefani Parmentier, Markus Schaich, Nikolas von Bubnoff, Frank Stegelmann, Angela Maurer, Martina Crysandt, Deniz Gezer, Maike Kortmann, Jeremy Franklin, Julia Frank, Martin Hellmich, Tim H. Brümmendorf, for the German Study Group for Myeloproliferative Neoplasms (GSG-MPN)

**Affiliations:** 1 Department of Medicine (Hematology, Oncology, Hemostaseology, and SCT), Faculty of Medicine, RWTH Aachen University, Pauwelsstr. 30, 52074 Aachen, Germany; 2 Center of Integrated Oncology Aachen Bonn Cologne Düsseldorf (CIO ABCD), Aachen, Germany; 3 Internal Medicine 3, Oncology, Hematology and Rheumatology, University Clinic Bonn (UKB), Bonn, Germany; 4 Internal Medicine V, Department of Hematology and Oncology, Comprehensive Cancer Center Innsbruck (CCCI), Medical University of Innsbruck, Innsbruck, Austria; 5 Hematology and Oncology, Jena University Hospital, Jena, Germany; 6 Internal Medicine C, Greifswald University Medicine, Greifswald, Germany; 7 Department of Oncology, Hematology, and Bone Marrow Transplantation With Section of Pneumology, University Medical Center Hamburg-Eppendorf, Hamburg, Germany; 8 Department of Hematology/Oncology, Klinikum Minden, Germany; 9 Department of Hematology/Oncology, Otto-Von-Guericke University Medical Center Magdeburg, Magdeburg, Germany; 10 Department of Internal Medicine 1, University of Dresden, Dresden, Germany; 11 Department of Medicine I – Hematology, Cell Therapy, Hemostaseology, University of Leipzig, Leipzig, Germany; 12 Department of Internal Medicine III, University Hospital of Ulm, Ulm, Germany; 13 Medical Department III, Hematology and Oncology, and Center for Translational Cancer Research, Translatum, Technical University of Munich, Munich, Germany; 14 Division of Clinical Oncology, Internal Medicine, Medical University of Graz, Auenbruggerplatz 15, 8036 Graz, Austria; 15 Department of Hematology, Oncology and Palliative Medicine, Rems-Murr Klinikum, Winnenden, Germany; 16 Oncology and Hematology, Tumor Center, St. Claraspital, 4058 Basel, Switzerland; 17 Department of Hematology, Oncology and Stem Cell Transplantation, Medical Center, Faculty of Medicine, Freiburg, Freiburg, Germany and German Cancer Consortium (DKTK), Partner Site, Freiburg, Germany; 18 Department of Hematology and Oncology, Medical Center, University of Schleswig-Holstein, Campus Lübeck, Lübeck, Germany; 19 Center for Translational & Clinical Research (CTC-A), Faculty of Medicine, RWTH Aachen University, Aachen, Germany; 20 Institute of Medical Statistics and Computational Biology, Faculty of Medicine and University Hospital Cologne, University of Cologne, Cologne, Germany

**Keywords:** Polycythemia vera (PV), Randomized clinical trial, Efficacy and safety, Ruxolitinib, Best available therapy, German Study Group for myeloproliferative neoplasms (GSG-MPN)

## Abstract

Patients (pts) with polycythemia vera (PV) suffer from pruritus, night sweats, and other symptoms, as well as from thromboembolic complications and progression to post-PV myelofibrosis. Ruxolitinib (RUX) is approved for second-line therapy in high-risk PV pts with hydroxyurea intolerance or resistance. The RuxoBEAT trial (NCT02577926, registered on October 1, 2015, at clinicaltrials.gov) is a multicenter, open-label, two-arm phase-IIb trial with a target population of 380 pts with PV or ET, randomized to receive RUX or best available therapy. This pre-specified futility analysis assesses the early clinical benefit and tolerability of RUX in previously untreated PV pts (6-week cytoreduction was allowed). Twenty-eight patients were randomly assigned to receive RUX. Compared to baseline, after 6 months of treatment, there was a significant reduction of median hematocrit (46 to 41%), the median number of phlebotomies per year (4.0 to 0), and median patient-reported pruritus scores (2 to 1), and a trend for reduced night sweat scores (1.5 to 0). JAK2V617F allele burden, as part of the scientific research program, also significantly decreased. One hundred nine adverse events (AEs) occurred in 24/28 patients (all grade 1 to 3), and no pt permanently discontinued treatment because of AEs. Thus, treatment with ruxolitinib in untreated PV pts is feasible, well-tolerated, and efficient regarding the above-mentioned endpoints.

**Supplementary information:**

The online version contains supplementary material available at 10.1007/s00277-022-05080-7.

## Introduction

Polycythemia vera (PV) is a myeloproliferative neoplasm (MPN) that is caused by somatic gain-of-function mutations in the *jak2 gene* in hematopoietic stem and progenitor cells [1], most of which result in the JAK2V617F oncoprotein [1]. Survival of patients (pts) with PV is decreased compared with age-matched controls [2], and this is mainly due to thromboembolic complications, or, in a smaller proportion of pts, progression to post-PV myelofibrosis, or even acute leukemia [3]. Strikingly, essentially all pts with PV report symptoms when they are systematically evaluated, i.e., using the MPN symptom assessment form (MPN-SAF) [4]. The most common symptoms include fatigue (91.7% of pts), insomnia (68.1%), numbness (66.2%), itching/pruritus (65.0%), sad mood (65.0%), early satiety (62.1%), and concentration problems (61.2%) [4]. Night sweats and bone pain are also present in about half of all PV patients (57.4% and 47.5%, respectively) [4]. Overall, 85.5% of PV pts report decreased quality of life [4]. Importantly, most of these symptoms persist even during complete hematologic remission (CHR) [5].

Treatment of PV is mainly directed at preventing thrombotic/thromboembolic events [6], and, based upon randomized trials [7] [8], currently, ASA and phlebotomies with a target Hct of < 45% are recommended for all PV patients [6]. In addition, cytoreductive therapy with hydroxyurea (HU) or ropeginterferon-alpha (ropegIFNa) is recommended for “high-risk” pts with PV, i.e., those aged > 60 years and/or with a history of thrombosis/thromboembolism [6], based upon a post hoc propensity-matched analysis of HU treatment in the prospective ECLAP cohort [9] and the randomized phase 3 trial [10] of ropegIFNa vs. HU treatment in such high-risk pts.

Two randomized phase 3 trials [11] have evaluated the role of the JAK inhibitor ruxolitinib (RUX) in PV pts who were intolerant or resistant to HU treatment and either had (RESPONSE trial [11]) or did not have splenomegaly (RESPONSE-2 trial [12]). In both studies, RUX was compared with best available therapy (BAT), which consisted of continued HU therapy in most pts. The results showed that RUX was superior to BAT in controlling Hct and improving PV-related symptoms [11], and in reducing spleen volume in those pts with splenomegaly [11]. Of note, although the study was not designed to formally address reductions in thromboembolic events, the RESPONSE trial did find a decreased rate of thromboembolic complications in the RUX- vs. BAT-treated group [13]. In addition, a retrospective real-world analysis of pts with HU-intolerant or HU-resistant PV treated with ruxolitinib or BAT found significantly less arterial thrombotic events in the RUX-treated group [14]. In a double-blinded comparison to hydroxyurea in PV patients who had been treated with HU before but still suffered from symptoms, RUX did not achieve the primary endpoint (> = 50% improvement of symptoms from baseline at week 16 of treatment) (43.4 vs. 29.6%, *p* = 0.139), but showed significant benefit in reducing pruritus [15]. Nevertheless, whether RUX treatment is also safe and effective in pts with untreated PV is currently unknown.

We report here the results from the futility analysis of the PV cohort treated within the phase 2b clinical trial entitled “Ruxolitinib versus best available therapy in patients with high-risk polycythemia Vera or high-risk essential thrombocythemia” (Ruxo-BEAT; NCT02577926) to assess the efficacy and tolerability of ruxolitinib in untreated PV pts.

## Methods

Ruxo-BEAT (NCT02577926) is a multicenter, open-label, two-arm phase 2b trial with a target population of 380 pts, divided into two strata of 190 untreated PV (a maximum of previous 6 weeks of HU, anagrelide, or interferon therapy was allowed) and 190 untreated or pretreated ET pts. All patients provided written informed consent, and the study was approved by the central ethics committee at RWTH Aachen University Hospital (EK 345/14) and at the local ethics committee of each center. The most relevant inclusion criteria were an age of 18 years of age or older, ECOG performance status of ≤ 2, a diagnosis of either PV or ET according to the WHO 2008 classification. Moreover, PV pts and ET pts had to be classified as high risk according to defined criteria as outlined below: for patients with high-risk PV or PV with indication for cytoreductive therapy due to progressive myeloproliferation, any one of the following had to be fulfilled ([16–18]): age > 60 years, previous documented thrombosis or thromboembolism, platelet count > 1500 × 10^9^/L, poor tolerance of phlebotomy or frequent phlebotomy requirement, symptomatic or progressive splenomegaly, severe disease-related symptoms (according to the local investigator’s definition), or progressive leukocytosis with leukocyte counts > 20 × 10^9^/L. The ET stratum of pts was not analyzed in the present futility analysis. Patients requiring prolonged use of oral corticosteroids with a dose of more than 20 mg per day (> 1 month) and patients with active splanchnic vein thrombosis within the last 3 months (included Budd-Chiari, portal vein, splenic and mesenteric thrombosis) were excluded. A detailed list of all in- and exclusion criteria can be found in the Supplementary Information.

Pts in each stratum were randomized in a 1:1 ratio to receive either RUX or best available therapy (BAT). Crossover from BAT to RUX was allowed in eligible patients after 6 months who did not achieve complete remission but still had sufficient hematologic reserves (platelet count ≥ 140 × 10^9^/L, hemoglobin ≥ 10.0 g/dL, and ANC ≥ 1.5 × 10^9^/L). Patients with PV in the RUX arm received a starting dose of 10 mg bid, which were allowed to be increased up to 20 mg bid due to insufficient efficacy. BAT included monotherapies of all commonly used medications (HU, anagrelide, interferon alpha, or others). BAT treatment was allowed to be changed during the course of the study. However, these patients were censored for primary endpoint analysis. The primary endpoint was the rate of complete clinico-hematologic response (CHR) at month 6 as defined by Barosi et al. Blood 2009 [19]. Secondary endpoints included the use of phlebotomy (recorded as the number of phlebotomies for the year before baseline and for 6 months during therapy, the latter of which was then calculated per year to be comparable with the pre-study phlebotomies), spleen size, patient-reported outcomes on specific MPN-associated symptoms using the MPN-SAF-TSS form (see appendix of the protocol), and overall survival. The MPN-SAF-TSS form is an abbreviated form of the former MPN-SAF questionnaire (9 out of 17 items) with the addition of one item from the so-called BFI questionnaire. The chosen items were the most clinically relevant items (concentration, early satiety, inactivity, night sweats, itching, bone pain, abdominal discomfort, weight loss, and fever) and in the MPN-SAF-TSS these items score on a 0 (absent/as good as it can be) to 10 (worst imaginable/as bad as it can be) linear analog self-assessment scale [20]. In the German version included in the present study, three additional symptoms regarding microvascular disturbances were added as well as a question on overall quality of life, while the question on fatigue and inactivity were combined.

The current futility analysis of PV patients randomized to RUX, after 50 PV patients overall had been enrolled, was pre-specified in the trial protocol in order to exclude early failure or excessive toxicity. Of these 50 patients, 28 patients were randomized into the RUX arm. Closure of the PV subgroup was pre-specified if no favorable trend were observed for any of the following variables: (1) improvement (decrease) in the hematocrit level during 6 months of treatment or (2) improvement of one of the following three symptom variables assessed by physician’s judgement or via MPN-SAF during 6 months of treatment: pruritus, night sweats, or bone pain. Furthermore, the change in JAK2V617F allele burden during the first 6 months of treatment was analyzed as part of the scientific research program. Differences between screening (Hct) or baseline (all other variables) and end of month 6 (EOM6) (all variables) were calculated using McNemar’s test (for physician-assessed pruritus and night sweats) or the Wilcoxon signed rank test (all other variables). Probability values of < 0.05 were considered significant.

## Results

### Baseline variables

Twenty-eight pts with untreated PV were included in this analysis. Baseline data of these pts are listed in Table 1. Median time from the first diagnosis to initiation of treatment in the context of the RuxoBEAT trial was 7.6 months (IQR: 1.5–29.2 weeks). The most common indications for treatment were age over 60 years and previously documented thrombosis or thromboembolism (each in 13 patients).

**Table 1:** Baseline characteristics of pts

Patients with untreated PV	*n* = 28
Mean age, in years (range)	60.6 (36–81)
Female patients, % (patients)	32 (9/28)
Caucasian Ethnicity, % (patients)	100 (28/28)
ECOG, % (patients)	
0	64 (18/28)
1	32 (9/28)
2 +	0 (0/28)
missing	4 (1/28)
Hct, median % (IQR) (screening)	46 (43–47)
Hemoglobin, median g/dl (IQR) (screening)	13.8 (12.5–15.2)
White blood count, median × 10^9^/L (IQR) (screening)	13.2 (8.1–17.1)
Platelet count, median × 10^9^/L (IQR) (screening)	503 (337–687)
Creatinine, mean mg/dl + / − SD	0.96 (0.17)
AST, mean U/L + / − SD	33.6 (16.95)
ALT, mean U/L + / − SD	34.8 (24.8)
Total Bilirubin, mean mg/dL + / − SD	1.00 (0.9)
Splenomegaly, % (patients)	57.7% (15/26; missing = 2)
Pre-Treatment (6 weeks) (% of pts)	25 (7/28)
Hydroxyurea, % (patients)	25 (7/28)
Anagrelide (%)	0 (0/28)
(Peg)interferon (%)	0 (0/28)
Pre-treatment duration (days; median, IQR)	12 (5.5–31.8)

^*^one pt. received HU and anagrelide simultaneously: this period was not counted twice; one case with missing dates

### Efficacy

At the time of data lock, 28 patients had received RUX for at least 6 months. Median time on drug during this first 6 months of treatment was 173 days (range 151–189). Efficacy variables at 6 months were assessed and compared to the screening/baseline variables, as pre-specified in the protocol for the futility analysis.

**Fig. 1:**
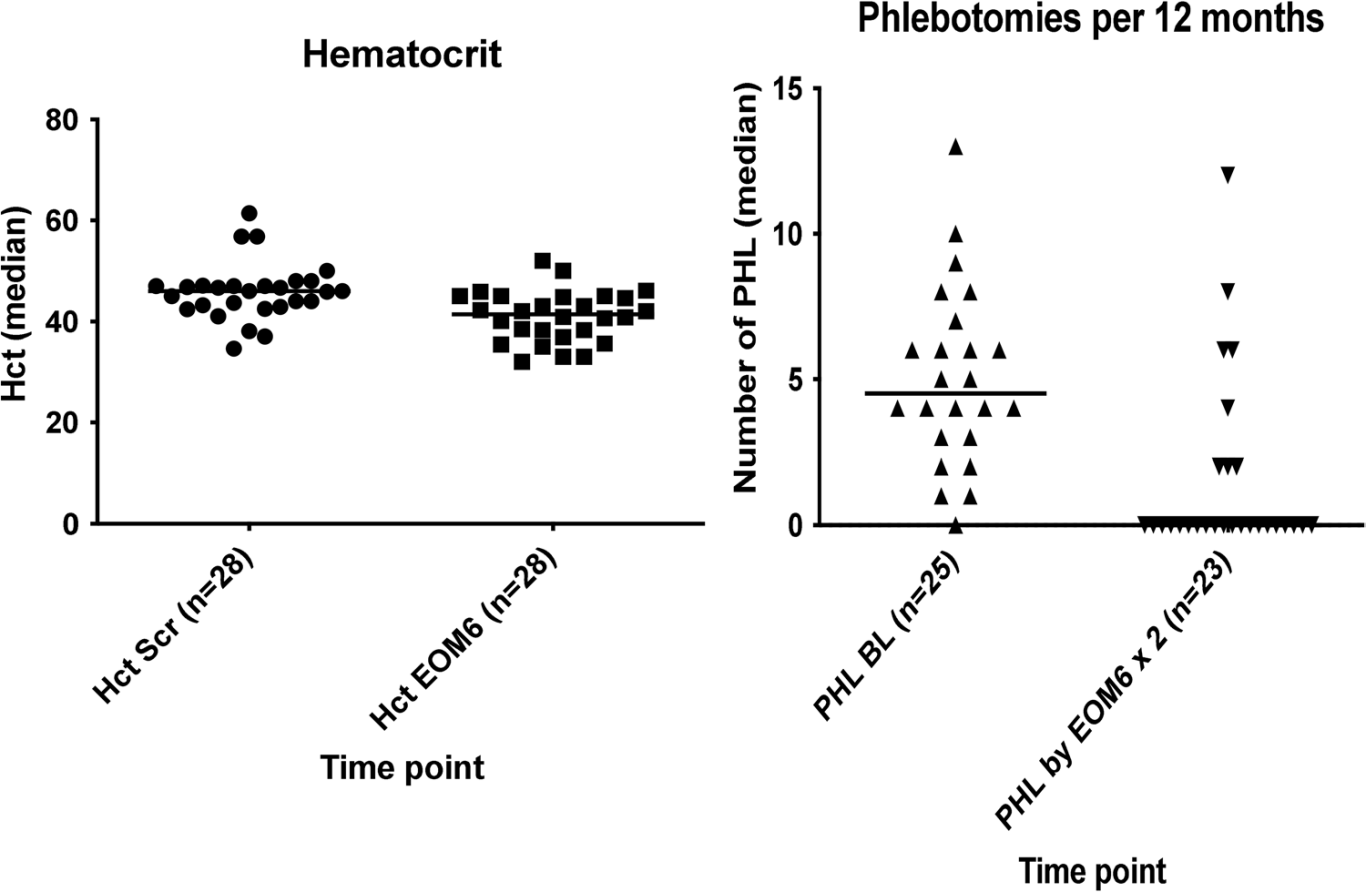
Hematocrit levels and frequency of phlebotomies over time. (Left) Hematocrit (Hct) was assessed at screening and at 6 months of RUX treatment (EOM6) and decreased from 46% (no missing values; range 34.6–61%) (IQR 42.98–47.08%) to 41.45% (no missing values; range 32–52%) (IQR 37.25–44.95%) (*p* = 0.000957). (Right) The number of phlebotomies (PHL) was assessed during 12 months prior to baseline (BL) and during 6 months of treatment (EOM6). In order to be able to compare both time points, the number of PHL was doubled to calculate the number per 12 months. The median number of phlebotomies performed per year decreased from 4.0 (no missing values; IQR 1.00–6.00) to 0 (missing = 3; IQR 0–2.0) (*p* = 0.000493)

**Fig. 2:**
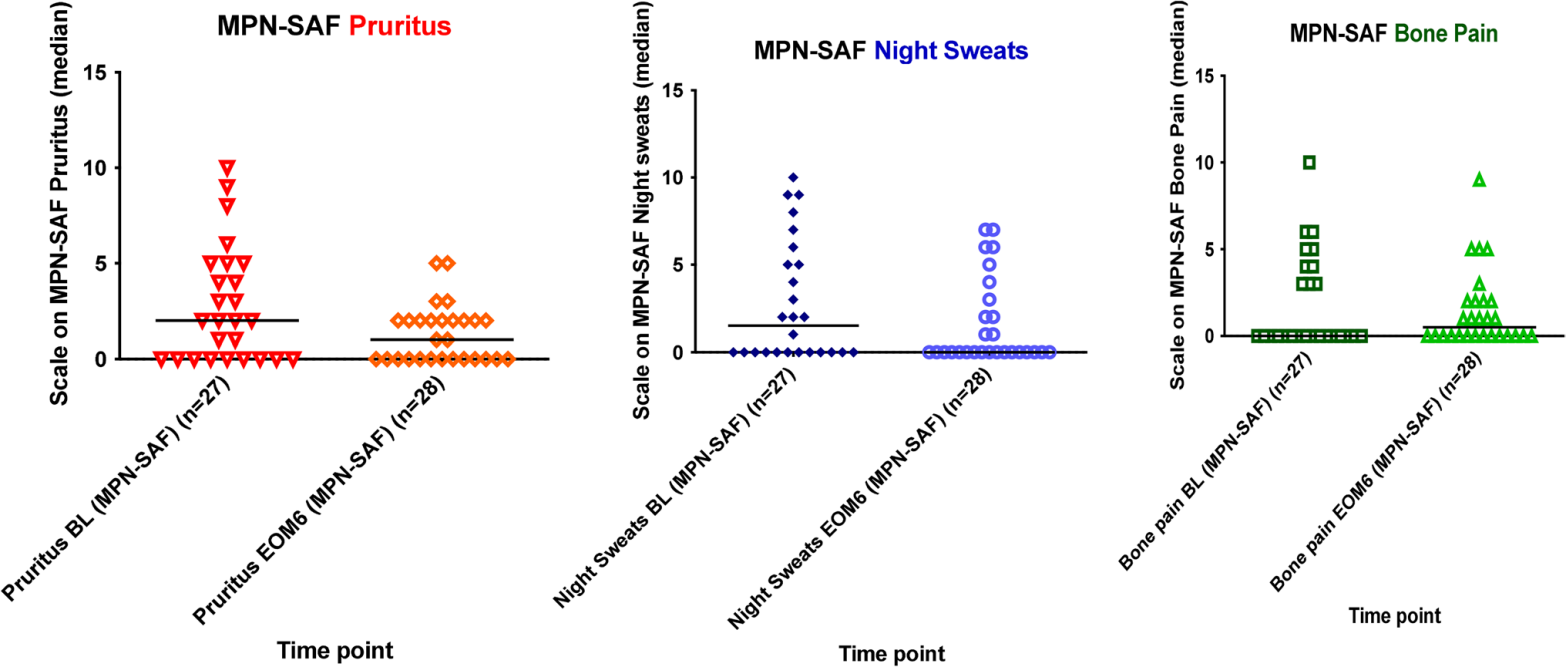
MPN-SAF symptom scores for pruritus, night sweats, and bone pain over time**.** Three symptoms (pruritus, night sweats, and bone pain) were assessed in patients at baseline (BL) and after 6 months of RUX treatment (EOM6), using the MPN-SAF questionnaire. The score for each individual patient is depicted. The median scores for pruritus decreased from median 2 (*n* = 26; missing = 2; range 0–10) (IQR 0–5) to 1 (range 0–5) (IQR 0–2) (*p* = 0.006), and a tendency for reduction was seen for the mean score of night sweats ((from median 1.5 (missing = 2; range 0–10) (IQR 0–5.25) to 0 (no missing values; range 0–7) (IQR 0–2.75); *p* = 0.101), while the mean score for bone pain remained unaltered (median 0 (missing = 2; range 0–10) (IQR 0–4) to 1 (no missing values; range 0–9) (IQR 0–2); *p* = 0.343)

After 6 months, the median hematocrit level decreased from 46% (no missing values; range 34.6–61%) (IQR 42.98–47.08%) to 41.45% (no missing values; range 32–52%) (IQR 37.25–44.95%) (*p* = 0.000957) (Fig. 1). The median number of phlebotomies performed per year decreased from 4.0 (no missing values; IQR 1.00–6.00) to 0 (missing = 3; IQR 0–2.0) (*p* = 0.000493) (Fig. 1). Median JAK2V617F allele burden decreased from 44% (missing = 5; range 16–93%) to 34% (missing = 5; range 9–91%) (*p* = 0.000973). The percentage of patients with pruritus or night sweats, as assessed by the physician, decreased from 36 to 14% (no missing values; *p* = 0.07), and from 29 to 11% (no missing values; *p* = 0.063), respectively. Patient-reported outcome points on the MPN-SAF survey for pruritus decreased from median 2 (*n* = 26; missing = 2; range 0–10) (IQR 0–5) to 1 (range 0–5) (IQR 0–2) (*p* = 0.006), and there was a tendency for reduction of night sweat points (from median 1.5 (missing = 2; range 0–10) (IQR 0–5.25) to 0 (no missing values; range 0–7) (IQR 0–2.75); *p* = 0.101), while the points for bone pain remained unaltered (median 0 (missing = 2; range 0–10) (IQR 0–4) to 1 (no missing values; range 0–9) (IQR 0–2); *p* = 0.343) (Fig. 2). The median MPN symptom score (mean of all point values from the 12 symptom-oriented questions) decreased from a median value of 1.5 (missing = 2; range 0.09–7) (IQR 0.87–3.0) to a median value of 1.14 (no missing values; range 0–6) (IQR 0.48–2.55) after 6 months of therapy (*p* = 0.061).

### Safety

During the first 6 months of therapy, 109 adverse events occurred in 24/28 patients. All of these AEs were grade 1 to 3, and no grade 4 or 5 events occurred. Eleven patients experienced at least one grade 3 AE, eight patients only grade 1 and 2 events, and five patients only experienced grade 1 events. Patients had a median of 3.0 events during the first 6 months of treatment (including the 4 without any such AEs). Of all 109 events, only 12 (11%) were grade 3, 31 (28%) were grade 2, and the majority (66/109 = 61%) were grade 1. Frequencies of AEs that occurred in more than 10% of the patients can be found in Table 2 (using system organ classes according to MedDRA level 14). The most frequent AE were general (fatigue), respiratory, dermatologic, renal and urinary, gastrointestinal, nervous system, cardiovascular disorders, infections, or musculoskeletal, connective tissue, or metabolic disorders (Table 2). Grade 3 AEs included two cases of liver enzyme increase, and one case each of compression fracture of spine with spinal canal stenosis, creatinine phosphokinase elevation, suspected coronary syndrome, urinary retention, herpes zoster, hot flashes, choroidal melanoma, macula edema, diabetic retinopathy, and hypertension.

**Table 2:** AEs occurring in > 10% of patients (i.e., at least in 3 patients)

Type of AE	% of pts (# of pts)	Number of events	Grade 1	Grade 2	Grade 3
General disorders and administration site conditions	35.7 (10)	18	13	5	None
Among these: Fatigue	21.4% (6)	9	6	3	None
Respiratory, thoracic, and mediastinal disorders	10.7 (3)	3	1	2	None
Skin and subcutaneous tissue disorders	25 (7)	8	7	1	None
Renal and urinary tract disorders	10.7 (3)	4	2	1	1 (1 case of bladder emptying disorder)
Gastrointestinal disorders	10.7 (3)	6	5	1	None
Nervous system disorders	25 (7)	10	7	3	None
Vascular disorders	14.3 (4)	7	3	2	2 (1 case of hot flashes, 1 case of hypertension)
Cardiac disorders	10.7 (3)	5	3	1	1 (1 case of acute coronary syndrome)
Infections	14.3 (4)	5	3	1	1 (herpes zoster)
Musculoskeletal and connective tissue disorders	14.3 (4)	8	5	2	1 (vertebral fracture)
Investigations	25 (7)	22	10	9	3 (2 cases of transaminase elevation, 1 case of creatinine phosphokinase elevation)
Metabolism and nutritional disorders	10.7 (3)	4	4	None	None

Fifty of all 109 AEs were deemed not to be related to the study treatment according to the treating physician. Relationship to ruxolitinib in the remaining adverse events was determined to be related (*n* = 11), likely (*n* = 3), possible (*n* = 35), or unlikely (*n* = 10). AEs were more likely to occur early during the course of treatment, with 68.8% of all events occurring during the first 3 months of therapy.

Only two patients had to interrupt treatment because of AEs (one case of irritability, one case of back pain). Both of them were re-exposed and subsequently remained on drug treatment without any dose reduction. Four dose reductions were due to AEs (one case each of elevation of transaminases, thoracic pain, hypoglycemia, and headache). No patient permanently discontinued treatment during 6 months of therapy.

Eight severe adverse events (SAEs) occurred in five patients. However, only one SAE was interpreted as clearly related to ruxolitinib (herpes zoster), another case was considered possibly related (thoracic pain), while all other SAEs were considered to be unrelated according to the treating physician.

### Disposition on treatment

All 28 patients began to study medication at the correct dose (10 mg BID). Within the first 6 months of study treatment, there were a total of seven dose reductions in six patients; two patients interrupted treatment because of AEs (see above). In addition, 11 dose escalations were reported in these patients.

None of the analyzed 28 randomized PV patients terminated the study within the first 6 months. There were no recorded protocol violations concerning informed consent, inclusion/exclusion criteria, or randomization.

## Discussion

In this futility analysis, we analyzed interim data to decide whether enrollment of pts with untreated PV into the randomized Ruxo-BEAT trial is safe and effective. The futility analysis showed significant improvements for Hct, JAK2V617F allele burden, and pruritus. Moreover, there was a significant reduction in the number of phlebotomies. Consequently, the trial was recommended to be continued by the Data Monitoring Committee.

RUX treatment was well-accepted by patients, as indicated by the lack of dropouts in the first 6 months of treatment among the patients analyzed so far. Also, there were no new safety signals of adverse events of RUX treatment, when compared to those already described in the RESPONSE [21] and RESPONSE-2 [12] trials and the trials involving pts with myelofibrosis [22–24]. Importantly, no grade 4 or 5 AEs occurred. The treating physicians rated 54% of the AEs as unrelated or unlikely related to RUX. Of eight SAEs, only two were deemed definitely or possibly related to RUX (herpes zoster and thoracic pain). Thus, RUX treatment was well-tolerated in pts with previously untreated PV. Given that a short period (6 weeks) of prior treatment with HU, anagrelide, or interferon was allowed, this might have had an influence on the efficacy. However, only 25%, 0%, and 0% of pts, respectively, received such treatment. And if these patients were excluded from the analysis, there was still a significant improvement of Hct (from median 46 to 40.6%, *p* = 0.002; no missing values) and JAK2V617F allele burden (from median 74.5 to 61.5%, *p* = 0.000973; missing *n* = 5) in the remainder of the pts.

Sustained control of hematocrit levels is of major importance in PV pts, in order to decrease cardiovascular mortality (death from cardiovascular reasons or major thrombotic events) [8]. Our results show that RUX is effective in reducing Hct levels, thereby potentially decreasing the risk for thromboembolic events (the 6-month endpoint was too early for thromboembolic event assessment) and that this was possible without phlebotomy support. This might also have a positive influence on the quality of life of the patients, given that patients requiring frequent phlebotomies may suffer from phlebotomy-induced symptomatic iron deficiency.

The continuous measurement of JAK2 allele burden during therapy is an interesting means of non-invasive disease monitoring, albeit not yet clinical routine, as the consequences of the results and guidelines on how to act on changes are still lacking. In the RESPONSE trial, significant decreases in JAK2 allele burden due to RUX treatment were reported [25], and pts with a more pronounced JAK2 allele burden reduction tended to show stronger spleen volume reductions [25]. However, no correlations with other clinical outcome variables were described. In our study, it will be interesting to analyze the correlations of the JAK2 allele burden with relevant clinical outcome data (such as thromboembolic complications or transformation to post-PV MF), given that the PV pts in this trial were mostly untreated and had been enrolled at an earlier disease timepoint, possibly rendering them more susceptible to JAKi treatment than the pts enrolled in the RESPONSE trials.

RUX significantly reduced pruritus in our cohort. This is an important finding, since pruritus in PV may severely reduce the quality of life and is often not sufficiently treated by other drugs [26]. Moreover, pruritus was present in more than 20% of ropeginterferon-treated pts [27]. Furthermore, in our trial, RUX also showed a strong trend towards reduction of patient-reported night sweats, while patient-reported bone pain, an additional common symptom in PV pts [4], scores remained unaltered. In the RESPONSE trial publication, this symptom has not been reported separately [21], although the MPN-SAF including this item was used in this trial. In pts with myelofibrosis, RUX did lead to a significant reduction in bone pain compared to placebo in the COMFORT-I-trial [22].

This pre-specified analysis has certain limitations: due to the short time of RUX treatment and the low patient number, we have not yet studied whether there was any effect on the rate of thromboembolism. This will be important to analyze at later time points during the trial. Also, more abundant information on complete blood counts and symptoms will then need to be analyzed. Finally, since it is important to assess how RUX treatment compares to BAT in this cohort of untreated PV pts, a comparative analysis will be performed in the interim analysis and the final analysis of the trial.

First-line treatment of PV is rapidly evolving, particularly with the recent approval of ropeginterferon, and it will be interesting to evaluate whether RUX proves beneficial in the first-line setting. Also, the question of optimal second-line treatment after ropeginterferon is currently unclear but may involve RUX treatment. Finally, while cytoreductive treatment in PV is not currently recommended for low-risk PV pts, Barbui and colleagues [28] have raised the question if also low-risk PV pts might benefit from early intervention. The results from the interim analysis of the randomized “LOW-PV” clinical trial, which compares standard treatment (phlebotomies and low-dose ASA) with standard treatment plus ropegIFNa in pts with low-risk PV, showed a significant benefit regarding the primary endpoint (defined as maintenance of the median hct values of ≤ 45% without progressive disease during a 12-month period) for the ropegIFNa group. Due to the superiority of the ropegIFNa arm, recruitment had to be stopped prematurely, and the 2-year-follow-up data are eagerly being awaited. Possibly, if the trial remains positive, pts with low-risk PV will soon be recommended to receive early cytoreductive treatment, and this may invoke the question of whether to give RUX or HU in the second-line setting in low-risk PV pts or, alternatively, to expand our study to low-risk pts.

In conclusion, the futility analysis of the RuxoBEAT trial confirms that treatment with RUX in untreated PV pts is effective (regarding the above-mentioned endpoints), feasible, and well-tolerated. The trial is currently ongoing.

## Supplementary information

Below is the link to the electronic supplementary material.

Supplementary file1 (DOCX 17.2 KB)

## Data Availability

Correspondence and requests for materials should be addressed to Steffen Koschmieder.
