# Supplementary material for: Efficacy and safety of ruxolitinib in patients with newly-diagnosed polycythemia vera: futility analysis of the RuxoBEAT clinical trial of the GSG-MPN study group
Source: Ann Hematol. 2022 Dec 23;102(2):349–58. doi: 10.1007/s00277-022-05080-7 (PMC9889492; doi:10.1007/s00277-022-05080-7)
Supplement: Supplementary file 1 — Supplementary file1 (DOCX 17.2 KB) [file 277_2022_5080_MOESM1_ESM.docx]

**Supplementary Information**

**List of inclusion/exclusion criteria:**

**Inclusion criteria**

1. Subjects must provide written informed consent prior to study-specific procedures or assessments which are not routinely performed for diagnosis or monitoring of PV or ET, and the subjects must be willing to comply with treatment and to follow up assessments and procedures

2. Patient must be 18 years of age or older

3. Patient´s ECOG performance status must be 0-2

4. Patient must fulfil WHO 2008 diagnostic criteria for either polycythemia vera (PV) or

essential thrombocythemia (ET). Moreover, PV- and ET-patients have to be classified as high risk according to defined criteria as outlined below.

- For patients with **high risk PV** OR **PV with indication for cytoreductive therapy due to progressive myeloproliferation**, AT LEAST ONE of the following must be fulfilled (according to DGHO onkopedia, (Barbui, et al., 2011), (Passamonti, 2009)):

• Age >60 years

• Previous documented thrombosis or thromboembolism

• Platelet count > 1500 x 109/L

• Poor tolerance of phlebotomy or frequent phlebotomy requirement

• Symptomatic or progressive splenomegaly

• Severe disease-related symptoms (according to the investigators definition)

• Progressive leukocytosis with leukocyte count > 20 x 109/L

- For patients with **high risk ET**, AT LEAST ONE of the following must be fulfilled (according to DGHO guidelines):

• Age > 60 years

• Platelet count> 1500 x 109/L

• Previous thrombosis or thromboembolism

• Previous severe hemorrhage related to ET (defined as decrease of Hgb of at least 2 g/dl)

5. Patients must fulfil the following criteria regarding prior therapy:

**PV patients:**

Never treated with cytoreductive drugs except hydroxyurea, anagrelide, or interferon for up

to 6 weeks maximum (phlebotomy and/or aspirin are allowed)

**ET patients**:

Naïve and pretreated patients may be entered in this trial.

6. Patient must have adequate liver function as indicated by a total bilirubin, AST, and ALT ≤ 2 of the institutional upper limit of normal (ULN) unless directly attributable to the patient’s MPN

7. Patient must have a creatinine clearance >40ml/min calculated according to the modified formula of Cockcroft and Gault, eGFR, or directly measured after 24h-urine collection

8. Patient must be able to swallow and retain oral medication

**Exclusion criteria**

1. Patients who meet criteria for post PV-MF or post ET-MF (IWG-MRT)

2. Patients who have received previous ruxolitinib treatment

3. Patients who have a history of anaphylaxis following exposure to the BAT drug of choice

4. Patients who have an inadequate bone marrow reserve as demonstrated by ANC ≤ 1 x 109/l OR platelet count <50 x 109/l

5. Patients who have known hepatitis B or C or HIV infection

6. Patients who suffer from other severe, concurrent diseases, including tuberculosis, serious cardiac functional dysfunction (class III or IV as defined by the New York Heart Association Classification), uncontrolled diabetes, uncontrolled hypertension, severe pulmonary disease (i.e. COPD with hypoxemia), or major organ malfunction that could interfere with the patient’s ability to participate in the study

7. Patients who have history of active substance or alcohol abuse within the last year

8. Female patients who are pregnant or nursing

9. Patients who have participated in another interventional trial and/or used investigational agents or concurrent anticancer treatment for concomitant disease within the last 4 weeks of registration

10. Any circumstance at the time of study entry that would preclude completion of the study or the required follow-up prohibits inclusion into this study

11. Subjects who have had an active malignancy during the previous 3 years except for treated cervical intraepithelial neoplasia, basal cell carcinoma of the skin, or squamous cell

carcinoma of the skin, each with no evidence for recurrence in the past 3 years

12. Patients who have uncontrolled bacterial, viral, or fungal infection

13. Patients who have any medical condition requiring prolonged use of oral corticosteroids with a dose of more than 20 mg per day (> 1 month)

14. Patients who have severe cerebral dysfunction and/or legal incapacity

15. Patients who have active splanchnic vein thrombosis within the last 3 months (includes Budd-Chiari, portal vein, splenic and mesenteric thrombosis)

16. Patients who have thyroid dysfunction which is not adequately controlled

17. Fertile men or women of childbearing potential cannot be included unless they are:

- surgically sterile or > 2 years after the onset of menopause and/or
- willing to use a highly effective contraceptive method (Pearl Index <1) such as oral contraceptives, intrauterine device, sexual abstinence, or barrier method of contraception (i.e. condoms) in conjunction with spermicidal jelly during study treatment

18. Patients who are taking any of the following prohibited medication:

- clarithromycin, telithromycin, troleandomycin (antibiotics)
- ritonavir, indinavir, saquinavir, nelfinavir, amprenavir, lopinavir (HIV protease inhibitors)
- itraconazole, ketoconazole, voriconazole, fluconazole (antifungals)

19. Patients with a diagnosis of galactose or lactose intolerance or a glucose-galactose-malabsortion
